# Supplementary material for: Sex Differences in the Prevalence of Chronic Pain in Mid-Life: A Systematic Review and Meta-Analysis
Source: Biomedicines. 2025 Oct 16;13(10):2523. doi: 10.3390/biomedicines13102523 (PMC12562057; doi:10.3390/biomedicines13102523)
Supplement: Supplementary file 1 [file biomedicines-13-02523-s001.zip › biomedicines-3806099-supplementary.pdf]

Table S1: Study protocol

Study protocol

Catherine Borra, Rebecca Hardy. Differences in chronic pain prevalence between men and women at mid-life: a systematic review. PROSPERO 2024 Available from https://www.crd.york.ac.uk/PROSPERO/view/CRD42021295895

REVIEW TITLE AND BASIC DETAILS

Review title

Differences in chronic pain prevalence between men and women at mid-life: a systematic review.

Review objectives

- What is the prevalence of CP in men and women in the general population at mid-life?
- What is the difference in CP prevalence between men and women in the general population?

Keywords

chronic pain, gender inequalities, health inequalities, persistent pain, prevalence, sex, sex inequalities

SEARCHING AND SCREENING

Searches

The selected databases are MEDLINE, to be accessed through Web of Science as an interface; and EMBASE, AMED and PsycINFO to be accessed through Ovid as an interface.

Study design

Study inclusion: Use any clearly stated CP definition in line with the International Association for the Study of Pain (IASP) definition of pain lasting longer than three months, including both local and widespread CP; Clearly state the country in which data was collected; Use data from an observational study, such as prospective and retrospective cohorts, cross-sectional and case control studies; Are written in English.
Study exclusion: Are reviews, conference proceedings, editorials and letters.

ELIGIBILITY CRITERIA

Condition or domain being studied

Epidemiological literature has revealed differences in chronic pain (CP) prevalence in men and women. Women are more likely to develop CP compared to men at different points of the life-course, like childhood and old age. Less is known about differences in prevalence during mid-life. While CP pools later in life, biological and physical changes in mid-life may predispose to an earlier differentiation in CP distribution – for example due to the menopause. The aim of this study is to describe the prevalence of CP at midlife in men and women, and to identify how these differences relate to results pertaining to other periods in the life-course.

Population

Inclusion: adults from the general population aged 40-60.
Exclusion: samples of specific groups, eg. clinical samples, population minorities.

Intervention(s) or exposure(s)

The exposure of interest is the presence of CP in line with the International Association for the Study of Pain (IASP) definition of pain lasting longer than three months, including both local and widespread CP.
Exclusion: studies specifically about neuropathic, diabetic or cancer pain.

Comparator(s) or control(s)

This si a prevalence study with no control arm.

OUTCOMES TO BE ANALYSED

Main outcomes

Estimates of CP; Estimates of sex difference; Estimates of CP prevalence for each sex.

Measures of effect

The primary estimates of interest are CP prevalence by sex and an estimate of the sex difference in pain (e.g. difference in prevalence or relative risk or odds ratio).

Additional outcomes

Not applicable.

DATA COLLECTION PROCESS

Data extraction (selection and coding)

After the database search is performed, duplicate search results will be removed from the final search list, which will be stored in Rayyan QCRI – a free systematic review software. Two reviewers will independently screen each title and abstract for eligibility using a template. The full text of the remaining articles will be retrieved using the UCL findit@UCL linking service. Inaccessible articles will be dealt with by contacting the authors directly. Each full text will be independently reviewed by two reviewers for final eligibility. Reasons for exclusion will be recorded. At each stage of screening, any differences between researchers will be resolved through discussion.
Data extraction will be conducted by the three reviewers for the following data items:
Citation details (including year of publication and title)
Study design
Country
Sample size
CP definition
CP location/type (local, widespread)
Measurement method for CP
Measurement method for age
Measurement method for sex/gender
Study type
Estimates of CP
Estimates of sex difference

Estimates of CP prevalence for each sex

A data extraction form will be used and data will be extracted independently for each paper by two of the three reviewers, who will resolve any discrepancies by discussion and supervision of an experienced member of the team (RH).

Risk of bias (quality) assessment

Study quality will be addressed using a tool for risk of bias assessment for prevalence studies which explores internal and external validity, and has previously been used in pain prevalence systematic reviews.

PLANNED DATA SYNTHESIS

Strategy for data synthesis

Narrative synthesis

A descriptive summary of studies will be provided using tables and addressing the following domains: primary outcomes, CP definition, CP type, sex/gender, age, geographic location (UN, WHO and HDI); and study quality assessment. It will comment on the integration of the methods used by the different studies and on the possibility for meta-analysis. The narrative synthesis will follow the Social Research Council Methods Programme guidelines (60), with a focus on identifying and exploring sources of heterogeneity.

Meta-analysis

A meta-analysis will be conducted if enough studies provide prevalence for the defined age group, and where the reviewers can justify combining results. A random effects meta-analysis will be used to combine estimates of CP prevalence by sex and a measure of difference in CP prevalence between sexes. These will be presented in a Forest plot. The I^2 will be used to assess the extent of heterogeneity in estimates.

Analysis of subgroups or subsets

Sub-group analysis or meta-regression will be performed to establish the extent of heterogeneity related to (i) geographic region (coded in three ways: UN, WHO and HDI), (ii) pain definition and (iii) pain type. Publication bias will be assessed separately using a funnel plot. A sensitivity analysis excluding low quality studies will be carried out.

REVIEW AFFILIATION, FUNDING AND PEER REVIEW

Review team members

- Ms Catherine Borra, University College London
- Professor Rebecca Hardy, University College London

Review affiliation

University College London

Funding source

Catherine Borra is supported by a PhD studentship funded by the Economic and Social Research Council (ESRC) and the Biotechnology and Biological Sciences Research Council (BBSRC) (award reference: ES\/T00200X\/1). Rebecca Hardy is Director of the CLOSER consortium, which is supported by the Economic and Social Research Council (ESRC) (award reference: ES\/K000357\/1).

Named contact

Catherine Borra

catherine.borra.19@ucl.ac.uk

TIMELINE OF THE REVIEW

Review timeline

Start date: 31 January 2022. End date: 30 June 2022

Date of first submission to PROSPERO

08 December 2021

Date of registration in PROSPERO

08 December 2021

Table S2: Search strategy. From: Borra, C. and Hardy, R. (2023) Chronic pain prevalence in men and women in mid-life: a systematic review. *BMJ Open, 13 (4).*

	MEDLINE (Web of Science)	*EMBASE + AMED + PSYCHinfo (Ovid)*
Pain terms	Chronic pain (MeSH Heading) OR fibromyalgia (MeSH Heading)<br>NOT<br>cancer OR diabetes OR neuropath* OR paed* OR child* OR adolescen*	Chronic pain OR persistent pain OR fibromyalgia (abstract)<br>NOT cancer OR diabetes OR neuropath* OR paed* OR child* OR adolescen* (abstract)
Study terms	epidemiology OR cohort stud* OR cohort analys* OR cross sectional stud* OR cross sectional analys* OR observational analys* OR prevalence OR disease frequency	Epidemiolog* OR cohort stud* OR cohort analys* OR cross sectional stud* OR cross-sectional* OR cross sectional analys* OR observational analys* OR prevalence OR disease frequency NOT trial OR clinical trial (abstract)
Moderators	Women OR female<br>Men OR male	AND Male OR men (all fields)<br>AND Female OR women (all fields)

Limits	Excluding RCTs and clinical studies/reviews<br>English language only<br>Journal articles only	English language only

Legend: MeSH terms are the Medical Subject Headings used for indexing articles in MEDLINE; The truncation command * is used to capture search terms which may have alternative endings; The Boolean logic operator AND combines results from the different search terms; The Boolean logic operator OR identifies results which include at least one of the search terms.

Table S3: Screening forms

**Bibliographic reference details:**			
First author			
Title			
Journal			
Volume			
Year of publication			
Reviewer	CB	JP	RH
Date			
**Inclusion**	Yes	No	
**Reasons for exclusion:**			
Ineligible population	Yes	No	
Ineligible study design	Yes	No	
Ineligible outcome	Yes	No	
Ineligible publication type	Yes	No	
Not in English	Yes	No	
Duplicate	Yes	No	
Other			

Table S4: Data extraction form

**Bibliographic reference details:**	
First author	
Title	

Journal				
Volume				
Year of publication				
Reviewer	CB	JP	RH	NR
**Study characteristics:**				
Study design	Cohort study	Cross-sectional study	Other:	
Sample size				
Country				
**Measurements:**				
CP definition	IASP	Other:		
CP measurement				
Sex measurement	Self-reported sex	Self-reported gender		
Age measurement				
**Outcomes:**				
Outcome type	OR	%	Other:	
Estimates of CP				
Estimates of sex difference				
Estimates of CP prevalence for each sex				
**Risk of bias:**				
External validity:				
Was the study's target population a close representation of the national population in relation to relevant variables?	Yes	No		

Was the sampling frame a true or close representation of the target population?	Yes	No
Was some form of random selection used to select the sample, OR was a census undertaken?	Yes	No
Was the likelihood of nonresponse bias minimal?<br>Internal	Yes	No
Were data collected directly from the subjects (as opposed to a proxy)?	Yes	No
Was an acceptable case definition used in the study?	Yes	No
Was the study instrument that measured the parameter of interest shown to have validity and reliability?	Yes	No
Internal validity:		
Was the same mode of data collection used for all subjects?	Yes	No
Was the length of the shortest prevalence period for the	Yes	No

parameter of interest appropriate?			
Were the numerator(s) and denominator(s) for the parameter of interest appropriate?	Yes		No
Summary item on the overall risk of study bias	Low	Moderate	High

Table S5: Study response rates, sampling frames and chronic pain case definition

**Study**	Sample approached	Analytical sample	Females (%), count	Response rate (%)	Chronic pain case definition	Data collection
**Andersson et al (1994)**	1806	1609	50.3 %	89.1%	Pain lasting over three months	Self-completed questionnaire
**Blyth et al (2001)**	24778 a	17543	57.1% b, 10012	70.8%	Pain experienced every day for three months in the six month period prior to interview	Interview
**Buskila et al (2000)**	2322	2210	60% b, 1326	95.2%	Chronic regional pain: Pain present on day of interview and had for longer than three months in one site; Chronic widespread pain: Pain present on day of interview and had for longer than three months according to 1990 American College	Interview

					of Rheumatology definition of fibromyalgia	
**Damsgård et al (2020)**	12455	5546	53.5 b, 2968 c	44.5%	Pain lasting for three months or more	Self-completed questionnaire
**Dominick et al (2011)**	Not known	12488	NK	67.9%	Pain that is present almost every day, but the intensity of the pain may vary. Pain that has lasted or is expected to last 6 months or more.	Self-completed questionnaire
**Elliott et al (2002)**	1937	1608	53.2% b, 855	83.0%	Pain present on day of interview and had for longer than three months.	Self-completed questionnaire
**Elzahaf et al (2016)**	1274	1212	51.3% b, 622	95.10%	Pain present on day of interview and had for longer than three months. Arabic version of the Structured Telephone Interview Questionnaire on Chronic Pain used in the Pain in Europe survey (2006) -	Telephone interview
**Jakobsson (2010)**	1800	826	58.6 %	48%	Pain lasting for three months or more	Self-completed questionnaire
**Johannes (2010)**	35718	27035	61.7% b, 16678	75.7%	Pain lasting for six months or more (chronic or recurring)	Self-completed questionnaire
**Kamerman (2020)**	12717	10336	59.3% b, 6126	81%	Pain lasting for three months or more (pain or discomfort)	Interview

**Mas (2008)**	2998	2192	Not known	Not known	1) widespread pain on the day of the interview, defined as a) pain in at least one point in both the right and the left half of the body, above and below the waist, and axial pain, b) which must be greater than one in a visual analogue scale (0-10), c) which had been present for more than three months, and d) which was unrelated to cancer or traumatism; and 2) finding of 11 out of 18 possible tender points on examination as established by the American College of Rheumatology (2011).	Interview and rheumatological assessment
**Miller (2017)**	20426	16412	Not known	84.8%	Pain lasting for six months or more (chronic or recurring)	Interview
**Raftery (2011)**	3136	1204	58.1% b, 699	40.1%	Current pain lasting for three months or more	Self-completed questionnaire
**Turhanoğlu (2008)**	Not known	600	50.7% b, 304	Not known	1) widespread pain on the day of the interview, defined as a) pain in at least one point in both the right and the left half of the	Self-completed questionnaire followed by clinical assessment

					body, above and below the waist, and axial pain, b) which must be greater than one in a visual analogue scale (0-10), c) which had been present for more than three months, and d) which was unrelated to cancer or traumatism; and 2) finding of 11 out of 18 possible tender points on examination as established by the American College of Rheumatology (2011).	
**White (1999)**	4674	3395	61.6% b, 2090	72.6% d	Pain for at least 1 week in past months in upper and lower quadrants lasting for longer than 3 months. Clinical assessment for all who screened positive using the fibromyalgia criteria from American College of Rheumatology (1990).	Interview
**Wolfe (1995)**	4018 e	3006	Not known	74.8%	Chronic pain: Pain present on day of questionnaire or interview and had for over three months.	Self-completed questionnaire

					Chronic widespread pain: Pain present on day of questionnaire or interview and had for over three months; and widespread as per the fibromyalgia criteria from American College of Rheumatology (1990).	
**Yeo (2009)**	9523	4141	57.8%	43.6%	Moderate to severe chronic persistent pain:<br>1) pain in the last 6 months,<br>2) of at least 3 months' duration,<br>3) of which 1 month was the month just prior to interview,<br>4) pain occurring several times a week,<br>5) pain was rated at least 4 and above on a 10-point verbal severity scale.	Interview
**a number not available in the study manuscript, derived by the researchers from the analytic sample size and response rate.**<br>**b percentage not available in the study manuscript, derived by the researchers from the analytic sample size and female count.**<br>**c female sample size was derived by the researchers using the female counts for Sami and non-Sami participants.**<br>d **percentage not available in the study manuscript, derived by the researchers from the percentage of non-responders.**<br>**e number not available in the study manuscript, derived by the researchers from the response rate and the analytic sample number.**						

Table S6: Study quality assessment and risk of bias, as per Hoy et al (2012)

	Andersson et al (1994)	Blyth et al (2001)	Buskila et al (2000)	Damsgård et al (2020)	Dominick et al (2011)	Elliott et al (2002)	Elzahaf et al (2016)	Jakobsson (2010)	Johannes et al (2010)	Kamerman et al (2020)	Mas et al (2008)	Miller et al (2017)	Raftery et al (2011)	Rustøen et al (2005)	Turhanoğlu et al (2008)	White et al (1999)	Wolfe et al (1995)	Yeo and Tai (2009)
Was the study's target population a close representation of the national population in relation to relevant variables?	Yes	Yes	Yes	Yes	Yes	No x	Yes	No 5	Yes	Yes	Yes	Yes	Yes	Yes	No 7	Yes	No 8	Yes
Was the sampling frame a true or close representation of the target population?	Yes	Yes	Yes	Yes	Yes	Yes	Yes	Yes	Yes	Yes	Yes	Yes	yes	Yes	Unclear	Yes	Yes	No 11
Was some form of random selection used to select the sample, OR was a census undertaken?	Yes	Yes	Yes	Yes	Yes	Yes	Yes	Yes	Yes	Yes	Yes	Yes	yes	Yes	Yes	Yes	Yes	Yes
Was the likelihood of nonresponse bias minimal?	Yes	Yes	Yes	No 3	No 4	Yes	Yes	No 3	Yes	Yes	Yes	Yes	No 3	No 3	Unclear	Yes	Yes	No 3
Were data collected directly from the subjects (as opposed to a proxy)?	Yes	Yes	Yes	Yes	Yes	Yes	Yes	Yes	Yes	Yes	Yes	Yes	Yes	Yes	Yes	Yes	Yes	Yes
Was an acceptable case definition used in the study?	Yes	Yes	Yes	Yes	Yes	Yes	Yes	Yes	Yes	Yes	Yes	Yes	Yes	Yes	Yes	Yes	Yes	Yes
Was the study instrument that measured the parameter of interest shown to have validity and reliability?	Yes	No 1	No 1	No 1	No 1	No 1	Yes	No 1	No 1	No 1	Yes	No 1	Yes	No 1	Yes	Yes	No 1	No 1
Was the same mode of data collection used for all subjects?	Yes	Yes	Yes	Yes	Yes	Yes	Yes	Yes	Yes	Yes	Yes	Yes	Yes	Yes	Yes	Yes	No 9	Yes
Was the length of the shortest prevalence period for the parameter of interest appropriate?	Yes	Yes	Yes	Yes	Yes	Yes	Yes	Yes	Yes	Yes	Yes	Yes	Yes	Yes	Yes	Yes	Yes	Yes
Were the numerator(s) and denominator(s) for the parameter of interest appropriate?	Unclear 2	Yes	Yes	Yes	Yes	Yes	Yes	Yes	Yes	Unclear 2	Unclear 6	Unclear 2	Yes	No 2	Yes	Yes	Unclear 2	Yes
Summary item on the overall risk of study bias	Moderate	Low	Low	High	Moderate	Low	Low	Moderate	Low	Moderate	High	Low	Low	High	High	Low	High	Moderate

Footnotes

1 No but similar questions for chronic pain definition have been used in research.
2 The study did not provide the sample sizes for males and females per each age group. However data from general analysis are appropriate.
3 Less than 50% response rate.
4 Moderate non-response bias, response rate 64%.
X The study analyses data from a general population postal survey, the geographic area is limited to the Grampian region of North East Scotland.
5 The study randomly recruited a sample from the Swedish Population Register and stratified in relation to age. The geographic area was limited to the province of Skåne.
6 The study did not provide the sample sizes different groups making it impossible to check results.
7 This was a cross-sectional population-based study. The geographic area was limited to the province of Diyarbakır.
8 The geographic area was limited to Wichita, Kansas.

9 Participants were contacted by both post and telephone.
10 The proportion of female respondents was higher compared to the 2005 Singapore census (P=0.047).

Table S7 Results from the supplementary meta-analysis of CP prevalence difference in females and males

	Studies	*Sample size*	*Pooled estimate*	*95% CI*	*I2*
Primary analysis					
	8	**17304**	0.05	0.03-0.07	31.15
Subgroup analysis by UN geographic region					
Africa	0	-	-	-	-
Asia	1	187	0.10	0.004-0.20	-
Europe	4	3465	0.07	0.04-1.11	0.01
Latin America and Caribbean	0	-	-	-	-
Northern America	2	7146	0.05	0.03-0.07	0.09
Oceania	1	6506	0.03	0.03-0.05	-
Subgroup analysis by risk of bias					
Low	5	14220	0.05	0.03-0.06	27.15
Moderate	1	160	0.06	-0.01-0.21	.
High	2	2924	0.08	0.04-0.11	0.01
Subgroup analysis by threshold for chronicity					
3 months	7	10617	0.06	0.03-0.08	37.15
6 months	1	6687	0.05	0.03-0.07	-
Subgroup analysis by pain type					
CP	6	16658	0.05	0.03-0.07	37.26
FIBROMYALGIA	2	646	0.06	0.03-0.09	0.02

Figure S1: Dot graph of relative risk of eligible studies.
The black circles (1.) represent the overall estimate in samples from Dominick et a; (2011), Elliott et al (2002), Jakobsson et al (2010), Johannes et al (2010), Kamerman et al (2020), Mas et al (2008, Raftery et al (2011), Rustoen et al (2005), White et al (1999) and Yeo et al (2009). Studies with estimates for different age groups are represented from lowest age group (1.) to highest (6.). These represent generic CP [Andersson et al (1994), Damsgard et al (2020), Elzahaf et al (2015), Mas et al (2008)] and FIBROMYALGIA [Turhanoglu et al (2008)]; CRP (1-2) and CWP (3-4)[(Buskila et al (2000)]; age groups in CRP (1-2), CWP (3-4) and FIBROMYALGIA (5-6) [Wolfe et al (1995))]; and very mild-to-mild and moderate-to-severe pain [Miller et al (2017)].

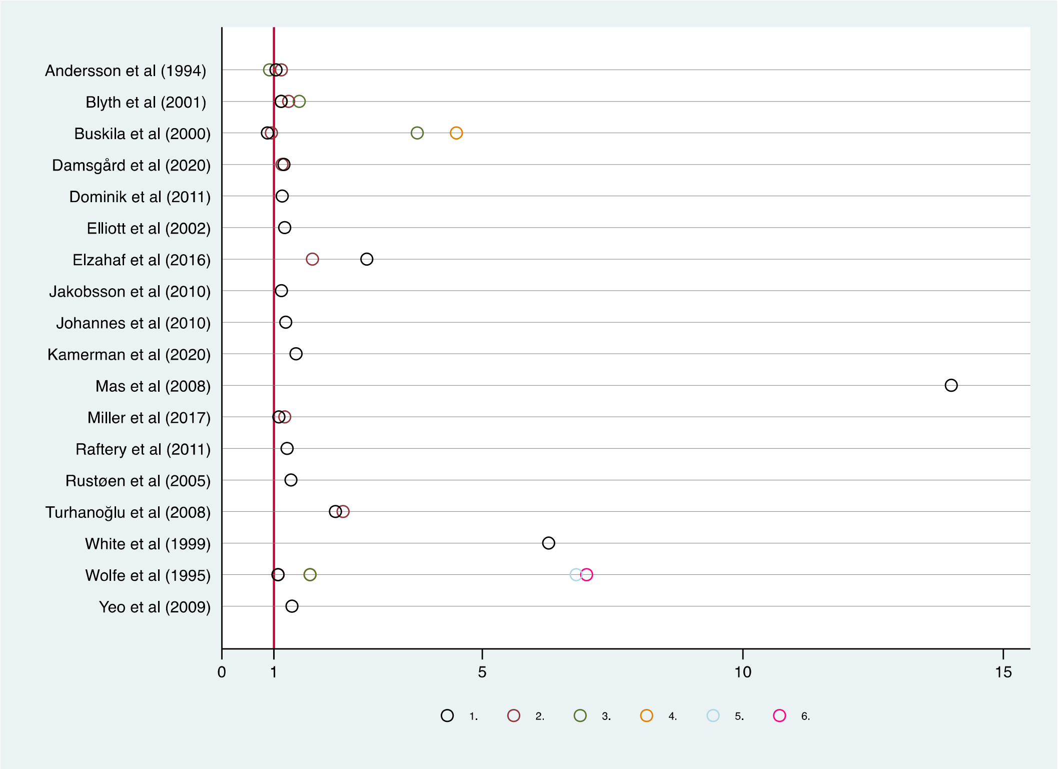

Andersson et al (1994)
Blyth et al (2001)
Buskila et al (2000)
Damsgård et al (2020)
Dominik et al (2011)
Elliott et al (2002)
Elzahaf et al (2016)
Jakobsson et al (2010)
Johannes et al (2010)
Kamerman et al (2020)
Mas et al (2008)
Miller et al (2017)
Raftery et al (2011)
Rustøen et al (2005)
Turhanoğlu et al (2008)
White et al (1999)
Wolfe et al (1995)
Yeo et al (2009)
0
1
5
10
15
1.
2.
3.
4.
5.
6.

Figure S2: Forest plot of sub-group analysis by geographic region

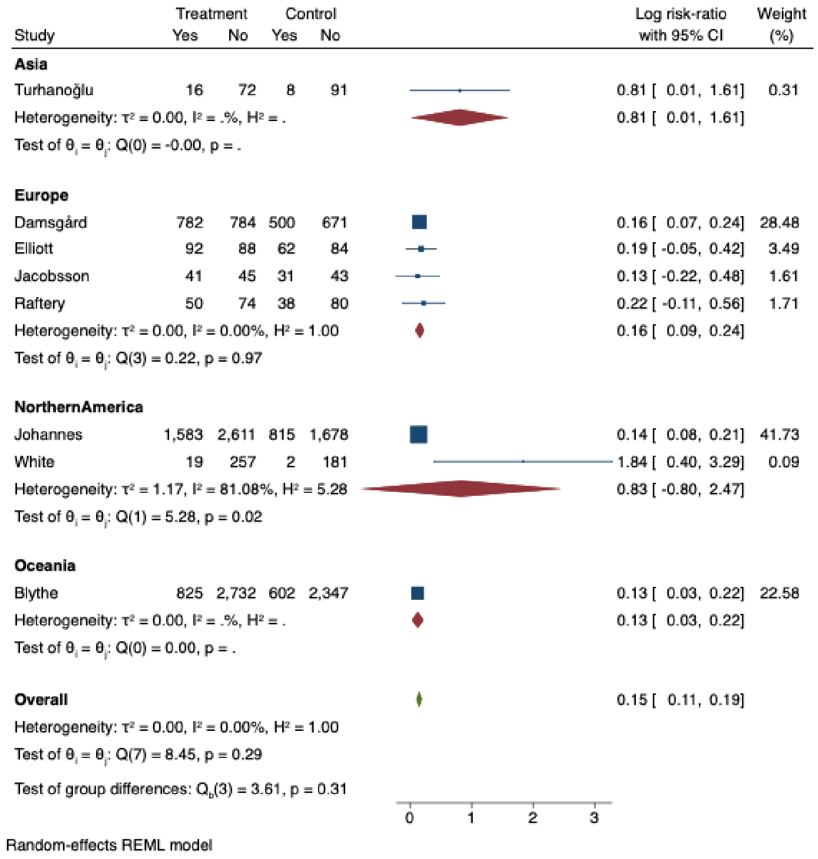

Figure S3: Forest plot of sub-group analysis by pain type

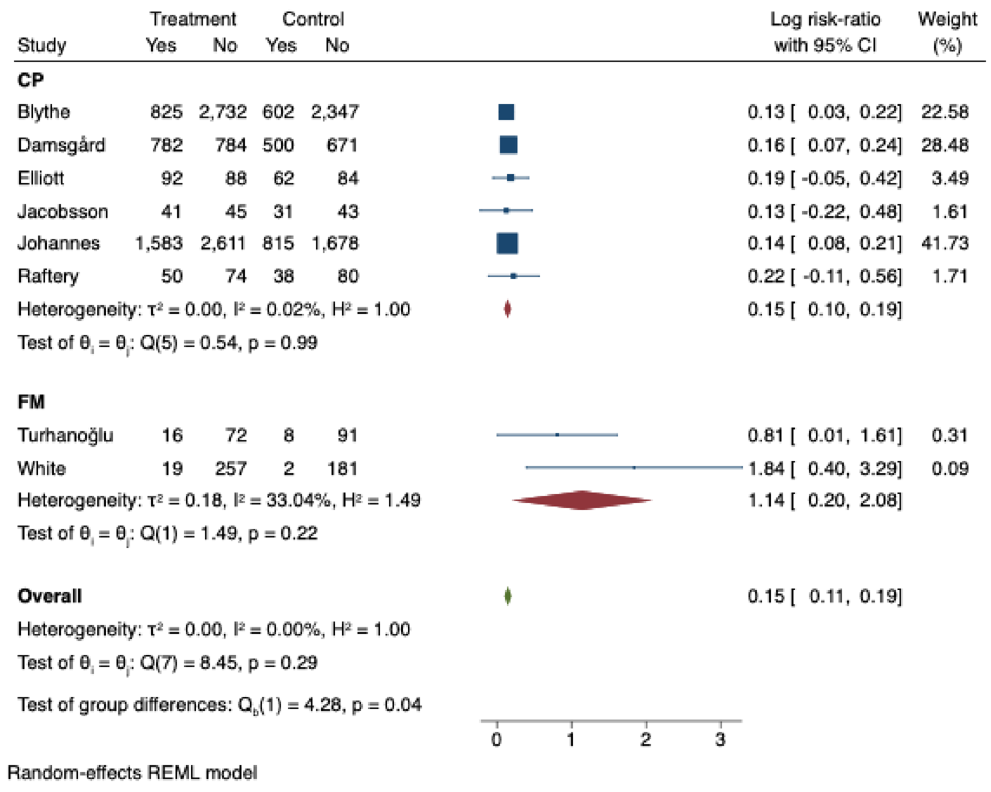

Figure S4: Forest plot for the meta-analysis of CP prevalence difference in females and males

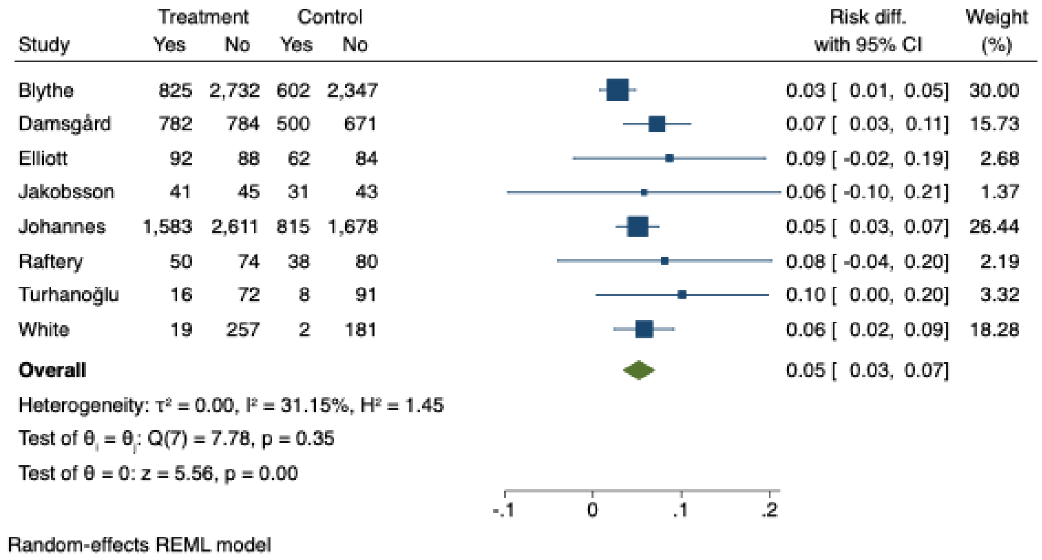

Figure S5: DOI plot for publication bias

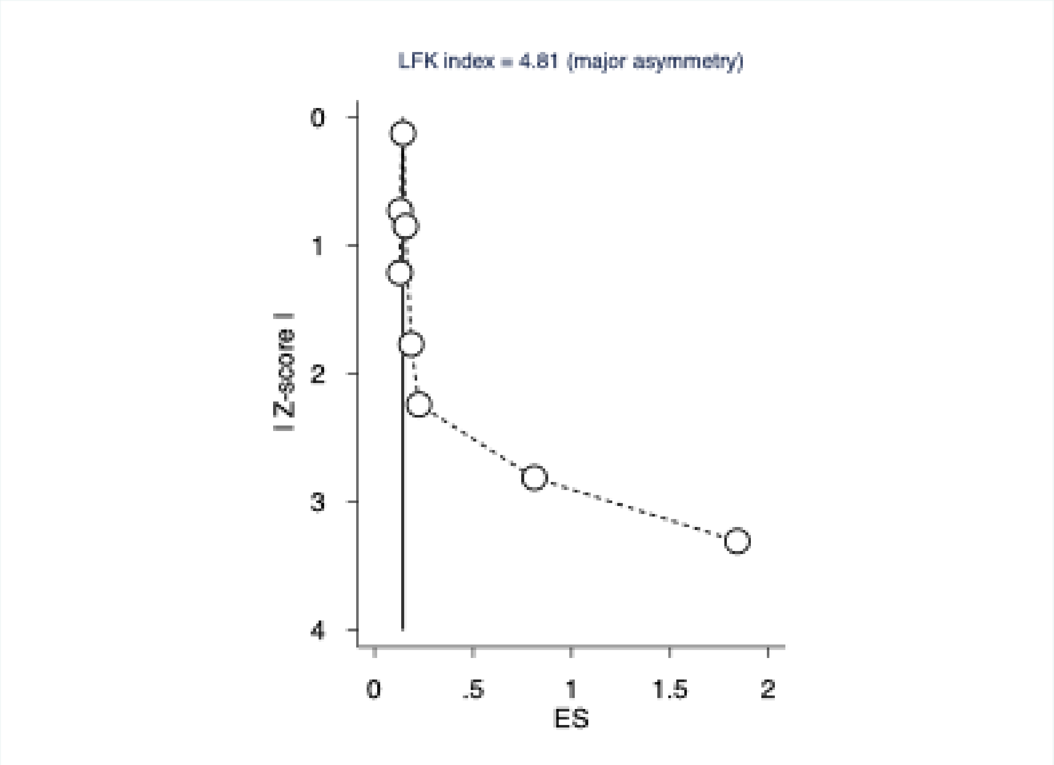

Table S8: PRISMA 2020 checklist

PRISMA 2020 Checklist

Section and Topic	Item #	Checklist item	Location where item is reported
**TITLE**			

Title	1	Identify the report as a systematic review.	P 1, line 2
**ABSTRACT**			
Abstract	2	See the PRISMA 2020 for Abstracts checklist.	P 1
**INTRODUCTION**			
Rationale	3	Describe the rationale for the review in the context of existing knowledge.	P 1-2, line 41-48
Objectives	4	Provide an explicit statement of the objective(s) or question(s) the review addresses.	P 2, line 58-61
**METHODS**			
Eligibility criteria	5	Specify the inclusion and exclusion criteria for the review and how studies were grouped for the syntheses.	P 3, line 73-80
Information sources	6	Specify all databases, registers, websites, organisations, reference lists and other sources searched or consulted to identify studies. Specify the date when each source was last searched or consulted.	P 3, line 67-71
Search strategy	7	Present the full search strategies for all databases, registers and websites, including any filters and limits used.	Appendix
Selection process	8	Specify the methods used to decide whether a study met the inclusion criteria of the review, including how many reviewers screened each record and each report retrieved, whether they worked independently, and if applicable, details of automation tools used in the process.	P3, line 82-92
Data collection process	9	Specify the methods used to collect data from reports, including how many reviewers collected data from each report, whether they worked independently, any processes for obtaining or confirming data from study investigators, and if applicable, details of automation tools used in the process.	P3, line 82-92
Data items	10a	List and define all outcomes for which data were sought. Specify whether all results that were compatible with each outcome domain in each study were sought (e.g. for all measures, time points, analyses), and if not, the methods used to decide which results to collect.	Appendix
	10b	List and define all other variables for which data were sought (e.g. participant and intervention characteristics, funding sources). Describe any assumptions made about any missing or unclear information.	Appendix
Study risk of bias assessment	11	Specify the methods used to assess risk of bias in the included studies, including details of the tool(s) used, how many reviewers assessed each study and whether they worked independently, and if applicable, details of automation tools used in the process.	P 3-4, line 94-109
Effect measures	12	Specify for each outcome the effect measure(s) (e.g. risk ratio, mean difference) used in the synthesis or presentation of results.	P 4, line 113-121
Synthesis methods	13a	Describe the processes used to decide which studies were eligible for each synthesis (e.g. tabulating the study intervention characteristics and comparing against the planned groups for each synthesis (item #5)).	P 10, line 179-189
	13b	Describe any methods required to prepare the data for presentation or synthesis, such as handling of missing summary statistics, or data conversions.	Table 2, Table 3
	13c	Describe any methods used to tabulate or visually display results of individual studies and syntheses.	N/A

	13d	Describe any methods used to synthesize results and provide a rationale for the choice(s). If meta-analysis was performed, describe the model(s), method(s) to identify the presence and extent of statistical heterogeneity, and software package(s) used.	P 4, line 113-121
	13e	Describe any methods used to explore possible causes of heterogeneity among study results (e.g. subgroup analysis, meta-regression).	P 4, line 113-121
	13f	Describe any sensitivity analyses conducted to assess robustness of the synthesized results.	P 4, line 113-121
Reporting bias assessment	14	Describe any methods used to assess risk of bias due to missing results in a synthesis (arising from reporting biases).	P 4, line 119-121
Certainty assessment	15	Describe any methods used to assess certainty (or confidence) in the body of evidence for an outcome.	N/A

PRISMA 2020 Checklist

Section and Topic	Item #	Checklist item	Location where item is reported
**RESULTS**			
Study selection	16a	Describe the results of the search and selection process, from the number of records identified in the search to the number of studies included in the review, ideally using a flow diagram.	P 5, figure 1
	16b	Cite studies that might appear to meet the inclusion criteria, but which were excluded, and explain why they were excluded.	P 5, figure 1
Study characteristics	17	Cite each included study and present its characteristics.	Table 1-2
Risk of bias in studies	18	Present assessments of risk of bias for each included study.	Table 1, supplementary materials B1-2
Results of individual studies	19	For all outcomes, present, for each study: (a) summary statistics for each group (where appropriate) and (b) an effect estimate and its precision (e.g. confidence/credible interval), ideally using structured tables or plots.	Table 1-2
Results of syntheses	20a	For each synthesis, briefly summarise the characteristics and risk of bias among contributing studies.	P 11, line 201-204; P 16, line 273-284
	20b	Present results of all statistical syntheses conducted. If meta-analysis was done, present for each the summary estimate and its precision (e.g. confidence/credible interval) and measures of statistical heterogeneity. If comparing groups, describe the direction of the effect.	P 14-17, line 239-298
	20c	Present results of all investigations of possible causes of heterogeneity among study results.	P 16,table 5
	20d	Present results of all sensitivity analyses conducted to assess the robustness of the synthesized results.	P 16-17, line 255-299

Reporting biases	21	Present assessments of risk of bias due to missing results (arising from reporting biases) for each synthesis assessed.	P 17, line 289-299
Certainty of evidence	22	Present assessments of certainty (or confidence) in the body of evidence for each outcome assessed.	N/A
**DISCUSSION**			
Discussion	23a	Provide a general interpretation of the results in the context of other evidence.	P 17-18, line 302-
	23b	Discuss any limitations of the evidence included in the review.	P 19, line 355-382
	23c	Discuss any limitations of the review processes used.	P 19, line 369-389
	23d	Discuss implications of the results for practice, policy, and future research.	P 19, line 394-395
**OTHER INFORMATION**			
Registration and protocol	24a	Provide registration information for the review, including register name and registration number, or state that the review was not registered.	P 3, line 63-65
	24b	Indicate where the review protocol can be accessed, or state that a protocol was not prepared.	P 3, line 63-65, appendix
	24c	Describe and explain any amendments to information provided at registration or in the protocol.	N/A
Support	25	Describe sources of financial or non-financial support for the review, and the role of the funders or sponsors in the review.	P 20, line 413-424
Competing interests	26	Declare any competing interests of review authors.	P 20, line 413-424
Availability of data, code and other materials	27	Report which of the following are publicly available and where they can be found: template data collection forms; data extracted from included studies; data used for all analyses; analytic code; any other materials used in the review.	Appendix

From: Page MJ, McKenzie JE, Bossuyt PM, Boutron I, Hoffmann TC, Mulrow CD, et al. The PRISMA 2020 statement: an updated guideline for reporting systematic reviews. BMJ 2021;372:n71. doi: 10.1136/bmj.n71

For more information, visit: http://www.prisma-statement.org/

Table S9: STROBE checklist

STROBE Statement—checklist of items that should be included in reports of observational studies

	Item No	Recommendation	Page	line
**Title and abstract**	1	(*a*) Indicate the study's design with a commonly used term in the title or the abstract	1	1-3
		(*b*) Provide in the abstract an informative and balanced summary of what was done and what was found	1	11-32
**Introduction**				
Background/rationale	2	Explain the scientific background and rationale for the investigation being reported	1	33-61
Objectives	3	State specific objectives, including any prespecified hypotheses	2	57-61
**Methods**				
Study design	4	Present key elements of study design early in the paper	1	1-3
Setting	5	Describe the setting, locations, and relevant dates, including periods of recruitment, exposure, follow-up, and data collection	NA	
Participants	6	(*a*) *Cohort study*—Give the eligibility criteria, and the sources and methods of selection of participants. Describe methods of follow-up<br>*Case-control study*—Give the eligibility criteria, and the sources and methods of case ascertainment and control selection. Give the rationale for the choice of cases and controls<br>*Cross-sectional study*—Give the eligibility criteria, and the sources and methods of selection of participants	3	72-80
		(*b*) *Cohort study*—For matched studies, give matching criteria and number of exposed and unexposed<br>*Case-control study*—For matched studies, give matching criteria and the number of controls per case		
Variables	7	Clearly define all outcomes, exposures, predictors, potential confounders, and effect modifiers. Give diagnostic criteria, if applicable	3	72-80
Data sources/ measurement	8*	For each variable of interest, give sources of data and details of methods of assessment (measurement). Describe comparability of assessment methods if there is more than one group	3	82-92
Bias	9	Describe any efforts to address potential sources of bias	3	82-92
Study size	10	Explain how the study size was arrived at	NA	
Quantitative variables	11	Explain how quantitative variables were handled in the analyses. If applicable, describe which groupings were chosen and why	3	82-92
Statistical methods	12	(*a*) Describe all statistical methods, including those used to control for confounding	4	111-121
		(*b*) Describe any methods used to examine subgroups and interactions	4	111-121
		(*c*) Explain how missing data were addressed	NA	
		(*d*) *Cohort study*—If applicable, explain how loss to follow-up was addressed<br>*Case-control study*—If applicable, explain how matching of cases and controls was addressed<br>*Cross-sectional study*—If applicable, describe analytical methods taking account of sampling strategy		
		(*e*) Describe any sensitivity analyses	4	111-121

Continued on next page

**Results**				
Participants	13*	(a) Report numbers of individuals at each stage of study—eg numbers potentially eligible, examined for eligibility, confirmed eligible, included in the study, completing follow-up, and analysed	5	Figure 1: Study identification flow diagram
		(b) Give reasons for non-participation at each stage	NA	
		(c) Consider use of a flow diagram	Figure 1: Study identification flow diagram	
Descriptive data	14*	(a) Give characteristics of study participants (eg demographic, clinical, social) and information on exposures and potential confounders	Table 1-2	
		(b) Indicate number of participants with missing data for each variable of interest	Table 1-2	
		(c) *Cohort study*—Summarise follow-up time (eg, average and total amount)		
Outcome data	15*	*Cohort study*—Report numbers of outcome events or summary measures over time		
		Case-control study—Report numbers in each exposure category, or summary measures of exposure		
		Cross-sectional study—Report numbers of outcome events or summary measures	Table 1-2	
Main results	16	(*a*) Give unadjusted estimates and, if applicable, confounder-adjusted estimates and their precision (eg, 95% confidence interval). Make clear which confounders were adjusted for and why they were included	Table 1-2	
		(*b*) Report category boundaries when continuous variables were categorized	NA	
		(*c*) If relevant, consider translating estimates of relative risk into absolute risk for a meaningful time period	Table 2-4	
Other analyses	17	Report other analyses done—eg analyses of subgroups and interactions, and sensitivity analyses	Table 5	
**Discussion**				
Key results	18	Summarise key results with reference to study objectives	17	302-307
Limitations	19	Discuss limitations of the study, taking into account sources of potential bias or imprecision. Discuss both direction and magnitude of any potential bias	19	369-389

Interpretation	20	Give a cautious overall interpretation of results considering objectives, limitations, multiplicity of analyses, results from similar studies, and other relevant evidence	19	392-395
Generalisability	21	Discuss the generalisability (external validity) of the study results	19	387-389
**Other information**				
Funding	22	Give the source of funding and the role of the funders for the present study and, if applicable, for the original study on which the present article is based	19	416-421

*Give information separately for cases and controls in case-control studies and, if applicable, for exposed and unexposed groups in cohort and cross-sectional studies.

Note: An Explanation and Elaboration article discusses each checklist item and gives methodological background and published examples of transparent reporting. The STROBE checklist is best used in conjunction with this article (freely available on the Web sites of PLoS Medicine at http://www.plosmedicine.org/, Annals of Internal Medicine at http://www.annals.org/, and Epidemiology at http://www.epidem.com/). Information on the STROBE Initiative is available at www.strobe-statement.org.

Table S10: MOOSE checklist

MOOSE Checklist for Meta-analyses of Observational Studies

Item No	Recommendation	Reported on Page No
Reporting of background should include		
1	Problem definition	1-2
2	Hypothesis statement	2
3	Description of study outcome(s)	2
4	Type of exposure or intervention used	3-4
5	Type of study designs used	4
6	Study population	3-4

Reporting of search strategy should include		
7	Qualifications of searchers (eg, librarians and investigators)	1
8	Search strategy, including time period included in the synthesis and key words	2, aappendix
9	Effort to include all available studies, including contact with authors	3-4
10	Databases and registries searched	3
11	Search software used, name and version, including special features used (eg, explosion)	3-4
12	Use of hand searching (eg, reference lists of obtained articles)	NA
13	List of citations located and those excluded, including justification	Fig 1
14	Method of addressing articles published in languages other than English	2
15	Method of handling abstracts and unpublished studies	2
16	Description of any contact with authors	NA
Reporting of methods should include		
17	Description of relevance or appropriateness of studies assembled for assessing the hypothesis to be tested	3
18	Rationale for the selection and coding of data (eg, sound clinical principles or convenience)	3
19	Documentation of how data were classified and coded (eg, multiple raters, blinding and interrater reliability)	3-4
20	Assessment of confounding (eg, comparability of cases and controls in studies where appropriate)	3-4
21	Assessment of study quality, including blinding of quality assessors, stratification or regression on possible predictors of study results	3-4
22	Assessment of heterogeneity	4
23	Description of statistical methods (eg, complete description of fixed or random effects models, justification of whether the chosen models account for predictors of study results, dose-response models, or cumulative meta-analysis) in sufficient detail to be replicated	4
24	Provision of appropriate tables and graphics	Tables 1-4, Figs 1-4, appendix

Item No	Recommendation	Reported on Page No
Reporting of results should include		
25	Graphic summarizing individual study estimates and overall estimate	Figs 1-4
26	Table giving descriptive information for each study included	Table 2
27	Results of sensitivity testing (eg, subgroup analysis)	Fig 3, Table 3
28	Indication of statistical uncertainty of findings	12-16
**Item No**	**Recommendation**	**Reported on Page No**
Reporting of discussion should include		
29	Quantitative assessment of bias (eg, publication bias)	16-17, Fig 4
30	Justification for exclusion (eg, exclusion of non-English language citations)	19
31	Assessment of quality of included studies	16-17, Fig 4, appendix
Reporting of conclusions should include		
32	Consideration of alternative explanations for observed results	18-19
33	Generalization of the conclusions (ie, appropriate for the data presented and within the domain of the literature review)	18-19
34	Guidelines for future research	18-19
35	Disclosure of funding source	19

From: Stroup DF, Berlin JA, Morton SC, et al, for the Meta-analysis Of Observational Studies in Epidemiology (MOOSE) Group. Meta-analysis of Observational Studies in Epidemiology. A Proposal for Reporting. *JAMA*. 2000;283(15):2008-2012. doi: 10.1001/jama.283.15.2008.
